# Supplementary material for: Prediction and real-time compensation of qubit decoherence via machine learning
Source: Nat Commun. 2017 Jan 16;8:14106. doi: 10.1038/ncomms14106 (PMC5241809; doi:10.1038/ncomms14106)
Supplement: Supplementary Information — Supplementary figures, supplementary discussion, supplementary methods and supplementary references. [file ncomms14106-s1.pdf]

SUPPLEMENTARY METHODS

Trapped ^{171}Yb^{+} ion qubits

The system considered in this work consists of a small, approximately homogeneous ensemble of trapped $^{171}\mathrm{Yb}^{+}$ ions in a linear Paul ion trap enclosed in an ultra-high vacuum (UHV) chamber, and controlled by an external microwave driving field in the presence of engineered dephasing noise. The ions are Doppler cooled on the $^2\mathrm{S}_{1/2}$ to $^2\mathrm{P}_{1/2}$ transition with a $369\,\mathrm{nm}$ laser tuned approximately $\gamma/2$ lower than the resonance frequency of the transition, where γ is the natural linewidth. Undesired states to which the ions may decay are depopulated using two additional repump lasers at $638\,\mathrm{nm}$ and $935\,\mathrm{nm}$. Details of the experimental system appear in [1].

Qubits are realised by the magnetic dipole transition in the ground state hyperfine splitting between the states $|0\rangle := {}^2\mathrm{S}_{1/2}\,|F=0, m_\mathrm{F}=0\rangle$ and $|1\rangle := {}^2\mathrm{S}_{1/2}\,|F=1, m_\mathrm{F}=0\rangle$ with a transition frequency of $12.6\,\mathrm{GHz}$ as shown in supplementary figure 1. State initialisation is accomplished using a 2.1 GHz sideband on the $369\,\mathrm{nm}$ laser to optically pump the ion state to the $|0\rangle$ state. The qubit transition is uniformly driven by an ultra-low phase noise vector signal generator (Agilent E8267D) locked to an atomic caesium (Cs) reference. The microwaves are injected into the trap as free-space waves directed through a conical microwave horn and a dielectric lens (Flann Microwave CL320-4901) which focuses the microwave field onto the ion cloud, see supplementary figure 1b.

Qubit measurement

The qubit state is read out by stimulating the $^2\mathrm{S}_{1/2}\,|F=1\rangle$ to $^2\mathrm{P}_{1/2}$ transition and detecting scattered photons using a photon multiplier tube and a single-photon counting module (Standford Research Systems, SR400). The signal is processed by the experiment computer to calculate an optimal prediction of the dephasing process to feedback to the signal generator in the next measurement cycle. All experimental measurement results are normalised to measurements of the dark state and bright state (achieved using a π rotation) immediately preceding the experiment, in order to account for fluctuations in laser intensity and ion number.

Phase shifts between the local oscillator and the qubits are measured via Ramsey spectroscopy, integrated over time T_R. After initialising the qubits in state $|0\rangle$, we apply two $\sigma_\mathrm{x}/2$ pulses separated by the Ramsey interrogation time, T_R, during which the qubits evolve freely in an equal superposition of the measurement basis states. The microwave frequency of the pulses is detuned from resonance ω_0 as dictated by the engineered noise. A detuning will result in a reduction of brightness tracing out the so-called Ramsey fringe whose centre can be approximated by a squared sine function, see supplementary figure 1c.

To measure this detuning from resonance, we carry out two sequential Ramsey interrogations with interleaved Doppler cooling and state initialisation, using an additional detuning of the carrier of $\pm 1/(4T_\mathrm{R})$, which corresponds to the full width at half maximum of the centre fringe. The difference in brightness detected between these two sequential measurements provides means to calculate the frequency/phase offset using trigonometric relations. This technique is called square-wave frequency modulation (SWFM) and is popular among the frequency metrology community [2]. Experimentally, the desired frequency shifts are realised using an external arbitrary waveform generator (Rigol DG4602), which produces a rectangular voltage waveform that is sent to the vector signal generator's external analogue FM port synchronous with the experimental protocol.

In our experiments a single realisation of this pair of Ramsey interrogations constitutes of a single measurement of duration T_M, including periods of system reinitialisation. While we employ an approximately homogeneous spatial ensemble of ions and single-shot detection over each Ramsey period, the definition of T_M is independent of the specific realisation of the measurement routine. In the case of a single ion giving binary detection events, T_M would simply be defined by the time taken to accumulate sufficient experimental results in order to deduce a temporal average value of the qubit state.

Dephasing noise engineering

The engineered dephasing noise in our experiments is realised as a time-varying frequency detuning of the system's local oscillator from resonance with the qubit transition, $\delta(t) \equiv (\Omega(t) - \omega_0)/\omega_0$, where ω_0 is the qubit frequency and $\Omega(t)$ is the noisy clock frequency. In the frame co-rotating with ω_0 this noise is indistinguishable from an external dephasing term in the Hamiltonian $\widehat{H}(t) = -\dot{\phi}(t)\,\widehat{\sigma}_\mathrm{z}/2$ where $\widehat{\sigma}_\mathrm{z}$ is the Pauli operator in the measurement basis, and the dot indicates a time derivative [3]. This noise thus produces phase evolution of the qubit relative to the local oscillator, $|\psi(t)\rangle = \frac{1}{\sqrt{2}}\left[|0\rangle + \exp^{-i\phi(t)}|1\rangle\right]$, with $\phi(t) = \int_0^t dt'\delta(t')$. The form of the time-varying noise signal is produced as outlined in [1] in order to produce an effective dephasing noise power spectral density with user-defined characteristics. This noise is generated by an external arbitrary waveform generator and applied to the microwave source using an additional analog FM input (see supplementary figure 1).

To ensure the dominance of the engineered noise over in-

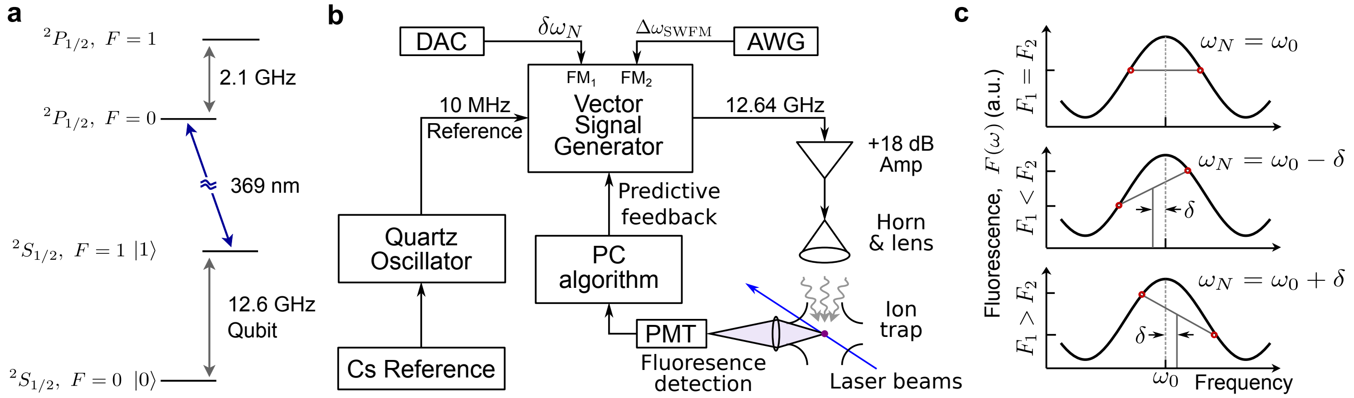

Supplementary Figure 1. Experimental setup and measurement technique. **a** Atomic energy level diagram of our trapped ^{171}Yb$^+$ ions (not to scale) showing the most important transitions . The qubit is realised through the hyperfine splitting of the $^1S_{1/2}$ ground state. Initialisation in the $|0\rangle$ state is accomplished using a 2.1 GHz sideband on the 369 nm laser, discriminative read-out between $|0\rangle$ and $|1\rangle$ by measuring scattered photons of the 369 nm laser without sidebands. **b** Simplified version of the experimental setup. We trap multiple ions in a linear Paul trap and drive the 12.6 GHz qubit transition with an ultra-low phase noise local oscillator locked to a 10 MHz reference. Engineered noise and SWFM shifts are applied using external FM. State detection and initialisation is accomplished using the 369 nm laser. The ion fluorescence is collected and focussed onto a photon multiplier tube whose signal is processed by a single-photon counting module. The resulting counts are processed by the experiment computer to provide optimal feedback to the LO. **c** Schematic procedure of square-wave frequency modulation (SWFM). The microwave frequency is shifted below and above the centre frequency of the microwaves by exactly $1/(4T_{\rm R})$. By measuring the difference in fluorescence at the two different frequencies we can calculate the difference between the centre frequency of the microwave source and the qubit transition frequency.

trinsic system noise, we scale its amplitude to be on the order of $20-50\,\%$ of the Ramsey fringe width. Previous demonstrations [1] have given coherence times of $T_2 > 2\,$s under intrinsic system conditions, while comparable phase accumulation here occurs on time scales of order tens of milliseconds.

Prediction estimation algorithm

The predictive algorithm employed here is derived from supervised machine learning and represents a simple yet efficient approach to optimisation problems with convex cost functions. We optimise the correlation between past measurements of qubit phase accumulation, $\phi^{\rm M}(t<t_k)$, under stochastic engineered dephasing noise, and future phase evolution of the qubit state, $\phi^{\rm M}(t>t_k)$. This is accomplished by constructing a linear combination of past measurements weighted by coefficients which are optimised with respect to the mean-squared error, ϵ, between our predictions and future measurement outcomes. This is equivalent to a multidimensional least-squares minimisation problem also known as linear regression, where the dimensionality is determined by the number of past measurements employed, n. While we possess quantitative knowledge of the engineered noise in our experiments, this information is not employed in any way in the optimisation routine and only serves to permit quantitative evaluation of predictive efficacy.

Weighting coefficients are calculated as a matrix $\mathbf{w} = \{w\}_{i,j}$ which produce a set of optimal predictions, $\phi^{\rm P}(t_{k+1}) = \sum_{i,j=0}^{n,k} w_{i,j}\phi^{\rm M}(t_j)$, for a range of past measurements, $i=1,..,n$, and discrete timesteps into the future, $j=1,..k$. We start with a set of initial weighting coefficients which are typically all zero except for the first one, i.e. the one relating to the most recent measurement, then calculate $\phi^{\rm P}$ and our cost-function $\epsilon = \|\ \mathbf{w}\cdot\boldsymbol{\phi}^{\rm M} - \boldsymbol{\phi}^{\rm P}\ \|^2$, where $\boldsymbol{\phi}^{\rm A,(M)} = \left\{\phi^{\rm A,(M)}\right\}_{i,j}$. A customised gradient-descent algorithm is employed to find the weights which minimise this cost-function for all i and j. Convergence is ensured by comparing the values of ϵ for sequential iterations and the speed of convergence is controlled by adaptive adjustment of the learning parameter. This algorithm was chosen as its performance scales favourably with matrix size, permitting efficient incorporation into real-time experimental demonstrations.

In order to ensure a conservative estimate of predictive performance we routinely determine the matrix $\mathbf{w}$ using a "training" data set that shares statistical properties with a target "validation" data set, but constitutes a different noise realisation. Proceeding in this manner ensures good predictive capabilities over an ensemble of similar noise realisations; such ensemble-averaged performance may be traded for enhanced predictive capabilities at the cost of diminished predictive robustness under different data sets. This tradeoff is commonly encountered in optimal control contexts [4] and may be referred to as "model robustness" or mitigation of "overfitting".

Time-division-multiplexed stabilisation

We craft a setting that replicates the constraints of quantum information experiments in which projective measurements are forbidden due to their role in causing quantum state collapse. We interleave periods of projective measurements

("probe") with periods in which the qubits undergo free evolution ("stabilisation") and are otherwise available for use in quantum information experiments. Our objective is to mitigate qubit dephasing during the stabilisation periods in which the qubit state is unsupervised, using information gained during the probe period and predictive estimation of future qubit dephasing.

During the probe period we perform n equally spaced measurements of the qubit's phase which are stored in memory and used to calculate $\phi^{\mathrm{P}}\left(t_k\right)$ for all t_k in the stabilisation period. The LO frequency is then corrected (effectively compensating for the qubit dephasing evolution) in each time step, t_k, up to a variable time at which a diagnostic measurement is performed. We vary $k = 1 \rightarrow 50$, with the entire measurement sequence including the probe period, prediction and step-wise stabilisation, repeated for each diagnostic experiment as k is varied. A general performance estimate is obtained by averaging over multiple probe and stabilisation periods.

Numerical simulations of these measurements are performed using the same engineered noise processes to which we add Gaussian noise with variance matching the expected shot noise level in our experiment. The simulations approximate the measurements well, indicating that they provide a good resource for performance estimates prior to experiments in our system.

Closed-loop predictive stabilisation

In this experiment we perform cyclic measurement feedback based on predictive estimation of future qubit phase evolution. Periodic measurements of the qubit-LO phase difference are performed and stored in memory on the experimental control computer. Using a previously calculated set of weighting coefficients $\mathbf{w}$ trained on a different noise realisation, we calculate $\phi^{\mathrm{P}}(t_k)$ at the time of correction in real time using the latest measurement outcomes. We then correct to compensate for the predicted phase offset, and measure the qubit state again. With each measurement we update our estimate of future evolution, feeding our algorithm the effectively uncorrected measurement by adding the correction to it. This way the correction is overlapping and continuous. This procedure is repeated over 1000 cycles in order to test the long-term stability of a qubit undergoing predictive stabilisation, and calculate the sample variance [5] as a function of the number of cumulative measurement cycles, N. The sample variance includes Bessel's correction in order to account for the finite nature of the measurement set. This routine is compared against a traditional feedback model in which corrections are cyclically applied based solely on the last measurement outcome without the use of any predictive estimation. Real-time feedback experiments are averaged over ten different noise realisations for both methodologies. We vary the effective sampling frequency of the measurement protocol relative to the spectrum of the engineered dephasing noise, $\omega_{\mathrm{s}}/\omega_{\mathrm{c}}$, by introducing gaps between measurements (skipping discrete timesteps) that correspond to "dead time" in precision frequency metrology.

The numerical simulations presented alongside with this data are obtained following the same overlapping protocol on the same noise realisations and approximate the experimental results well.

SUPPLEMENTARY DISCUSSION

The experiments presented in the main text employ quasi-white engineered noise spectra with fixed cut-off frequencies, ω_{c}, selected for experimental convenience and the ease of quantitative analysis. Here we provide evidence that the approach we employ to predict qubit stochastic dephasing is broadly applicable across a range of noise spectra, and sampling rates.

Complex coloured noise

We provide numeric simulations of the performance of our predictive algorithm in the context of closed-loop predictive feedback stabilisation, taking the same approach as for the experiments and simulations presented in figure 3 in the main text. We calculate the resulting sample variance of the free-running and stabilised qubit-LO system employing three different power spectra of dephasing noise:

- $1/\omega$ power spectrum with a white floor up to a high-frequency cutoff,
- Power spectrum including multiple different colours of noise, spanning from ω^2 to $1/\omega^2$,
- $1/\omega$ power spectrum with narrow spurs as may be encountered in synthesisers subject to interference from mains power or mechanical pickup.

In all cases we calculate the sample variance at 1000 stabilisation cycles, varying the sampling rate ω_{s} as expressed in terms of the (arbitrary) maximum frequency, $\omega_{\max}$ used in the power spectra. As before, a varying sampling rate is simulated by increasing the step size between two successive points in the time-domain realisation. Calculated performance is shown in supplementary figure 2, normalised to the sample variance of the free-running system. Predictive stabilisation is compared against traditional feedback, revealing order-of-magnitude improvements at the lowest sample rates, and demonstrating gains of tens of percent for $\omega_{\mathrm{s}} \rightarrow \omega_{\max}$. This behaviour appears because a reduction in the sampling frequency (dead time increases) increases aliasing of the noise and has detrimental impact on traditional feedback. Notably the greatest advantages appear for predictive estimation in cases where there is substantial noise power near the inverse cycle time [5]. We also observe that in cases where aliasing leads to destabilisation of the locked qubit-LO system under

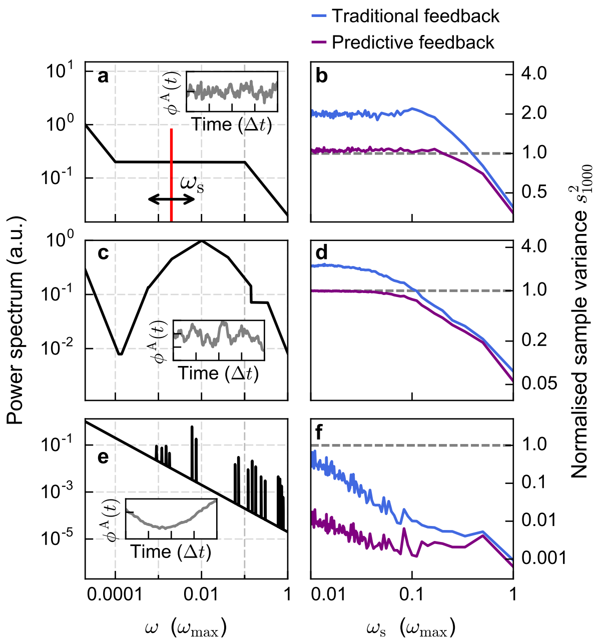

Supplementary Figure 2. Analysis of the efficacy of our approach to minimising LO-qubit dephasing for three different power spectra. **a, c, e** Various power spectra in arbitrary units with maximum frequency $\omega_{\max}$ normalised to 1. Examples include a $1/\omega$ spectrum with white floor ranging from $0.0001 - 0.1\,\omega_{\max}$, a complex coloured spectrum spanning from $1/\omega^2$ to ω^2 and a $1/\omega$ spectrum with spurs at frequencies ranging from $0.0001 - 0.8\,\omega_{\max}$. Insets show sample time-domain representations. The vertical line in the top left panel illustrates the change of sampling frequency in the analysis. Any frequency in the power spectrum greater than half this value is aliased. **b, d, f** Sample variance of the corrected data relative to their associated free-running oscillators (grey dashed line). Both left and right panels are relative to the same maximum frequency. These calculations use $n = 50$ past measurements.

traditional feedback, the predictive algorithm learns that the best strategy is to apply no correction, leading the predictive scheme to converge to the sample variance of the free-running system.

Intrinsic system noise

In addition to the quantitative performance demonstrations of our algorithm on engineered noise, we provide a validation of its efficacy on the native noise in our experiment. The underlying intrinsic instability of our system is dominated by a combination of phase noise inherent to the local oscillator and ambient magnetic field drifts, leading to a complex temporal form of measured qubit dephasing. Measurement shot noise has the added effect of increasing the amplitude of the flat noise floor of our measurements and reducing the quality of correlations that can be extracted from the data.

We calculate the performance of predictive feedback and traditional feedback control at a large number of cycles based on measurements of the free-running qubit-LO system. The small size of the residual phase/frequency instabilities in our system mandate a relatively long Ramsey time ($T_{\rm R} = 60\,$ms), and technical limitations impose an approximately $1.7\,$Hz sampling rate. We collect over 15,000 sequential measurement and use the first $70\,\%$ of the data set for training of the weighting coefficients in $\mathbf{w}$ and the remainder for validation.

The calculated sample variance at 5000 cycles of simulated feedback demonstrates that while the high frequency nature of noise in the system leads traditional feedback to destabilise the locked qubit-LO system, the use of $n > 40$ past measurements in calculating optimal prediction demonstrates improvements of up to $30\,\%$ relative to the free-running system. Gains relative to traditional feedback are commensurately higher.

This demonstration reveals that the real-time implementation of predictive feedback control can, in principle, provide substantial improvements under realistic conditions using state-of-the-art qubit-LO systems. Further improvements may be gained by reducing latency in the control cycle through use of dedicated high-speed FPGA hardware, instead of a control PC, for both data acquisition and the calculation of predictions in real time.

SUPPLEMENTARY REFERENCES

[1] Soare, A. and Ball, H. and Hayes, D. and Zhen, X. and Jarratt, M. C. and Sastrawan, J. and Uys, H. and Biercuk, M. J., "Experimental bath engineering for quantitative studies of quantum control," *Phys. Rev. A*, vol. **89**, p. 042329, 2014.

[2] Gill, P., ed., *Frequency standards and metrology, proceedings of the 6th symposium*. Singapore: World Scientific Publishing Co. Pte. Ltd., 2002.

[3] Ball, H. and Oliver, W. D. and Biercuk, M. J., "Upper-bounds on qubit coherence set by master clock instabilities," *arXiv*, vol. 1602.04551 [quant-ph], 2016.

[4] Stengel, R. F., *Optimal control and estimation*. Mineola, New York: Dover Publications Inc., 1994.

[5] Sastrawan, J. and Jones, C. and Akhalwaya, I. and Uys, H. and Biercuk, M. J., "Analytically exploiting noise correlations inside the feedback loop to improve locked-oscillator performance," *Phys. Rev. E*, vol. **94**, p. 022204, 2016.
